# The sound of lichens: ultrasonic acoustic emissions during desiccation question cavitation events in the hyphae

**DOI:** 10.1093/jxb/erae318

**Published:** 2024-07-24

**Authors:** Enrico Boccato, Francesco Petruzzellis, César Daniel Bordenave, Andrea Nardini, Mauro Tretiach, Stefan Mayr, Fabio Candotto Carniel

**Affiliations:** Department of Life Sciences, University of Trieste, via L. Giorgieri 10, 34127, Trieste, Italy; Department of Life Sciences, University of Trieste, via L. Giorgieri 10, 34127, Trieste, Italy; Instituto ‘Cavanilles’ de Biodiversidad y Biología Evolutiva (ICBiBE), Botánica, Fac. CC. Biológicas, Universitat de València, 46100 Burjassot, Valencia, Spain; Department of Life Sciences, University of Trieste, via L. Giorgieri 10, 34127, Trieste, Italy; Department of Life Sciences, University of Trieste, via L. Giorgieri 10, 34127, Trieste, Italy; Department of Botany, University of Innsbruck, Sternwartestraße 15, 6020 Innsbruck, Austria; Department of Life Sciences, University of Trieste, via L. Giorgieri 10, 34127, Trieste, Italy; Ghent University, Belgium

**Keywords:** Cavitation, chlorophyll *a* fluorescence, desiccation, lichen, LTSEM, turgor loss point, ultrasonic acoustic emission, water status dynamics

## Abstract

Lichens are a mutualistic symbiosis between a fungus and one or more photosynthetic partners. They are photosynthetically active during desiccation down to relative water contents (RWCs) as low as 30% (on dry mass). Experimental evidence suggests that during desiccation, the photobionts have a higher hydration level than the surrounding fungal pseudo-tissues. Explosive cavitation events in the hyphae might cause water movements towards the photobionts. This hypothesis was tested in two foliose lichens by measurements of ultrasonic acoustic emissions (UAEs), a method commonly used in vascular plants but never in lichens, and by measurements of PSII efficiency, water potential, and RWC. Thallus structural changes were characterized by low-temperature scanning electron microscopy. The thalli were silent between 380% and 30% RWCs, when explosive cavitation events should cause movements of liquid water. Nevertheless, the thalli emitted UAEs at ~5% RWC. Accordingly, the medullary hyphae were partially shrunken at ~15% RWC, whereas they were completely shrunken at <5% RWC. These results do not support the hypothesis of hyphal cavitation and suggest that the UAEs originate from structural changes at hyphal level. The shrinking of hyphae is proposed as an adaptation to avoid cell damage at very low RWCs.

## Teaser

Ultrasonic acoustic emissions and LTSEM observations of desiccating lichens do not support the hypothesized occurrence of explosive cavitation events in fungal hyphae as triggers of water movement.

## Introduction

Lichens are defined as symbioses between a fungus (the mycobiont) and at least one photoautotrophic organism, usually green algae and/or cyanobacteria (the photobiont) (Honegger, 2009), and they can harbour a diverse microbiota, including other algae, fungi, and bacteria (Hawksworth and Grube, 2020). They are poikilohydric organisms, meaning that their water content varies depending on the availability of liquid water and the water vapour pressure of the environment (Lange *et al*., 1997). Most lichens are desiccation tolerant, meaning that they can survive complete desiccation down to a water content <0.1 g H_2_O g^–1^ on a dry mass basis (Holzinger and Karsten, 2013) and rehydrate and reactivate their metabolism within a few minutes to hours when water is available again (Lidén *et al.*, 2010). Once completely desiccated, they enter a state called anhydrobiosis, in which their metabolism is completely shut down (Candotto Carniel *et al.*, 2021). In this state, lichens are among the most extremotolerant organisms. Accordingly, they can be found from deserts to high-alpine and polar environments, and can cope with challenging environmental conditions (Kranner *et al.*, 2008). The growth of lichens in environments where water availability is scarce or highly variable also highlights their excellent ability to exploit the potentially limited periods when thallus hydration and light availability ensure a positive CO_2_ balance (Nash *et al.*, 1982; Lange *et al*., 2007; Gasulla *et al.*, 2021). Indeed, there is direct (e.g. CO_2_ uptake) and indirect (e.g. Chl *a* fluorescence) evidence that lichens can still photosynthesize at low rates even when the water content of the thallus falls below 40–30% on a dry mass basis (Tuba *et al.*, 1996; Bednaříková *et al.*, 2020; Barták *et al.*, 2021). An illustrative example is the epilithic *Protoparmeliopsis muralis*, whose net photosynthesis time was only 16.7% of a year (Lange, 2003). During most of this time on a typical day (excluding periods after rain events), photosynthesis was rarely at its peak, because when the temperature rose in the morning, the lichens also began to lose water, so that they dried within a few hours. This situation has been described for many lichen species that thrive under similar water availability conditions (e.g. Lange *et al*., 1977; Schroeter *et al.*, 1992; Phinney *et al.*, 2018; Solhaug *et al.*, 2021). For this reason, lichens are exposed to continuous cycles of desiccation and rehydration, which limit the total CO_2_ gain but also allow the exchange of substances (e.g. nutrients dissolved in the hydration water) with the environment and between the two symbionts (Honegger, 1997).

In most studies dealing with the phenomena related to desiccation/rehydration processes, it is generally assumed that the mycobiont and the photobiont have the same hydration level (e.g. Csintalan *et al.*, 1998; Bukhov *et al.*, 2004; Green *et al.*, 2011). However, experimental evidence suggests that the photobiont layer has a higher hydration level during desiccation compared with the surrounding fungal pseudo-tissues. By studying water relation parameters in lichens, Nardini *et al.* (2013) proposed that photobionts have a significantly different osmotic potential and cell wall elasticity from the mycobiont hyphae, and that this may play a role in the water relations and water exchange between the two symbionts. Petruzzellis *et al.* (2018) combined the study of water relation parameters with measurements of Chl *a* fluorescence on the lichen *Flavoparmelia caperata* (L.) Hale and its isolated and cultivated photobiont *Trebouxia gelatinosa* Archibald, and found that in the lichen the water potential (Ψ) at turgor loss point (Ψ_tlp_) occurred at lower Ψ values than in the isolated photobiont. They also found that the maximum quantum yield (*F*_v_/*F*_m_) of PSII started to decrease significantly at Ψ values lower than Ψ_tlp_ in both thalli and isolated photobionts, and that, importantly, *F*_v_/*F*_m_ in thalli decreased at lower Ψ values than in non-lichenized photobionts. Therefore, assuming that *F*_v_/*F*_m_ can be used as a proxy for Ψ of the photobiont within the thallus, it was hypothesized that the photobiont layer had a higher hydration level during desiccation than the surrounding fungal pseudo-tissues, ensuring charge separation in PSII even at very low thallus water contents. Despite the robust evidence based on thermodynamic parameters, a mechanism explaining this phenomenon has not been described yet. Potkay *et al.* (2020), who developed a mechanistic model of water and vapour transport within *F. caperata* thalli, suggested that the different hydration level between the two symbionts during desiccation could be due to higher resistances of the cortices to vapour flow compared with the medulla, with water transfer from the medulla to the photobiont layer, allowing for prolonged PSII activity. Another possible mechanism underlying this phenomenon was proposed by Scheidegger *et al.* (1995). Using low-temperature scanning electron microscopy (LTSEM), they observed that in thalli of *Lobaria pulmonaria* (L.) Hoffm. and *Pseudevernia furfuracea* (L.) Zopf. at relative water contents (RWCs) below 85% and 60%, respectively, a considerable number of cortical and medullary hyphae had an internal globular cavity estimated to occupy >50% of the protoplast volume, which was absent in fully hydrated cells. They interpreted these cavities as gas bubbles resulting from explosive cavitation events in the fungal hyphae and suggested that cavitations could cause a ‘short apoplasmic water pulse’ from the cortical and medullary hyphae towards the photobiont layer, thus delaying photobiont desiccation (Scheidegger *et al.*, 1995). These gas bubbles were also detected by TEM and LTSEM in various lichen species (Honegger, 1995; Honegger *et al.*, 1996; Kappen *et al.*, 1996).

In vascular plants, cavitation events can occur under drought stress when a gas phase is aspirated in the xylem water column under negative hydrostatic pressure, via air-seeding at the inter-conduit pit membranes. Once in the xylem conduit, the gas phase expands explosively, releasing energy and filling the conduit with a gaseous embolism (Zimmermann, 1983). Hence, from a physical point of view, drought-induced embolism in vascular plants is not triggered by spontaneous cavitation of water under tension (Nardini *et al.*, 2024). In comparison, lichen hyphae can withstand very negative Ψ values, a necessary condition for ‘pure’ cavitation events to occur (Honegger *et al.*, 1996; Bastmeyer *et al.*, 2002), but they contain a living protoplast, a condition very different from that of xylem conduits which are dead at maturity and simply lined by the cell wall. In the past years, cavitation events in vascular plants have been frequently monitored to study desiccation processes by detecting the ultrasonic acoustic emissions (UAEs) generated by the expansion of the gas phase (e.g. Tyree and Dixon, 1983; Salleo *et al.*, 2000; Mayr *et al.*, 2006; Simbeye *et al.*, 2023; Nardini *et al.*, 2024).

The monitoring of UAEs is non-destructive, can be performed during desiccation, and has never been used on lichens, but only on non-lichenized fungi (Phillips *et al.*, 2023). Therefore, UAE measurements could be a viable option to gain new insights into the hypothesized origin of cytoplasmic gas bubbles from cavitation events proposed by Scheidegger *et al.* (1995). In this study, we recorded UAEs produced by lobes of the lichens *F. caperata* and *L. pulmonaria* during desiccation and coupled them with measurements of Ψ, water content, *F*_v_/*F*_m_, and LTSEM observations to assess whether (i) *F*_v_/*F*_m_ significantly decreases at more negative Ψ values than Ψ_tlp_, indicating delayed turgor loss in the photobiont and thus potential water shifts from the fungal partner; (ii) lichens emit UAEs with characteristics consistent with the energy released by explosive cavitation events; and (iii) the UAE peak occurs at RWC values close to Ψ_tlp_, supporting the hypothesis that cavitation-induced water movements occur in the thalli and delay Ψ drop of the photobionts.

## Materials and methods

### Thalli collection, preparation, and conservation

Thalli of *F. caperata* were collected from ash (*Fraxinus ornus* L.) trees in the Classic Karst plateau (Gabrovizza, 45°44ʹ0″N, 13°44ʹ0″E), while thalli of *L. pulmonaria* were collected from beech (*Fagus sylvatica* L.) trees in a wood of the Carnic Alps (Sauris Lake, 46°26ʹ54.01″N, 12°43ʹ21.04″E). Thalli without necrosis or damaged portions were detached from trunks (circumference ≥30 cm; height from the ground ≥60 cm) using a stainless cutter, placed in paper bags, and transported to the laboratory. Thalli were air-dried for 48 h at 20 °C, 55% relative humidity, 10 µmol m^–2^ s^–1^, 10/14 h light/dark cycle, and then cleaned from bark and moss fragments using stainless steel tweezers under a dissecting microscope. Thereafter, thalli were dried over silica gel for 48 h, vacuum sealed in plastic bags, stored at –20 °C for up to 3 months, and thawed over silica gel for 48 h before use.

### Sample preparation

Marginal lobes of different thalli (hereafter referred to as samples) of ~2–3.7 cm and weighing 88.2 ± 25.9 mg (*n*=31 per species) were excised and randomly selected for the experiments. Samples were pre-treated to ensure homogeneous hydration and minimize potential photo-inhibition due to differences in irradiance at the collection sites. Samples were soaked in deionized water for 3 min, blotted on adsorbent paper to remove excess water, and placed in plastic boxes with deionized water at the bottom and lined with wet adsorbent paper to maintain high relative humidity (>95%). The boxes were closed, but not sealed, with transparent film, in order to ensure gas exchanges, and kept at controlled conditions in a climatic chamber (20.0 ± 1.5 °C, 12/12 h light/dark cycle, 23 ± 1 µmol s^–1^ m^–2^). The hydration procedure was repeated three times a day (morning, afternoon, and evening) for 2 d to maintain samples fully hydrated. At the end, some of the wet samples (*n*=8 per species) were devitalized by placing them in a laboratory oven at 70 °C for 2 h into a Petri dish containing wet paper to keep them wet during the heat shock treatment (Tretiach *et al.*, 2012).

On the third day, living and devitalized samples were dark adapted for 30 min (Jensen, 2002) and then Chl *a* fluorescence (Chl_*a*_F) measurements were carried out using a Handy PEA fluorometer (Plant Efficiency Analyser, Hansatech Instruments Ltd, Norfolk, UK) to assess the PSII functionality as a proxy of the sample health (Bussotti *et al.*, 2011). Measurements were performed using a saturation pulse of 1500 μmol m^–2^ s^–1^ for 0.8 s and a ×0.8 gain factor. Steady-state (*F*_0_) and maximum fluorescence (*F*_m_) were used to calculate the variable fluorescence (*F*_v_=*F*_m_–*F*_0_) and the maximum quantum yield of PSII, *F*_v_/*F*_m_.

### Measurements of water potential isotherms and Chl_*a*_F

Since we expected cavitation phenomena to occur at a water potential (Ψ) close to the turgor loss point (Ψ_tlp_), Ψ isotherms (i.e. the relationship between Ψ and the volume of water lost by the samples) were measured on living samples following Petruzzellis *et al.* (2018). To monitor the joint *F*_v_/*F*_m_ decrease induced by turgor loss, Chl_*a*_F was measured on the same samples as a proxy of the photobiont water status inside the thallus. Specifically, six samples of each species were pre-treated as described above, rehydrated one last time, blotted with adsorbent paper, and weighed to record the FW. Then, samples were progressively desiccated at laboratory conditions (20 °C, 55% relative humidity) in the dark, and sequential measurements of Ψ, Chl_*a*_F, and FW were performed over time.

Ψ measurements were carried out using a dewpoint hygrometer (WP4C, METER Group Inc., Pullman, WA, USA). The instrument was calibrated at the beginning of each measurement session with a 0.5 M KCl solution, which corresponds to a Ψ of –2.19 MPa (22 °C). Samples were placed in the sample holder of the WP4C and kept open to allow water loss. The sample holder was then closed and left equilibrating for 30–45 min until stable Ψ values were obtained (±0.05 MPa). After each Ψ measurement, samples were removed from the holder while keeping them in the dark, placed in a standard Handy-PEA leaf clip, and Chl_*a*_F was measured as previously described. Subsequently, the sample FW was measured using an analytical balance. This process was repeated several times, until *F*_v_/*F*_m_ zeroed. The samples were then oven-dried for 24 h at 80 °C to measure the DW.

### Elaboration of water potential isotherms

The water relation parameters Ψ_tlp_ and the RWC at turgor loss point (RWC_tlp_) were determined using the Ψ isotherms. Since WP4C has a relatively high degree of uncertainty for Ψ values close to 0 and to overcome a possible influence by oversaturation due to apoplastic water, Ψ values from 0.00 MPa to –0.30 MPa were excluded from the analysis. Below this range of values, a decrease of FW corresponded to a significant decrease of Ψ, which means that all apoplastic water is lost (Nardini *et al*., 2013; Petruzzellis *et al*., 2018). The first FW after the loss of apoplastic water was then considered as the initial fresh weight (IFW). Values of water loss (WL) were calculated as follows:


$$\begin{array}{l} WL_{i}=IFW-FW_{i}, \end{array}$$

where WL_i_ is the water loss at the ith given time during desiccation, IFW is the initial fresh weight of the sample at full turgor, and FW_i_ is the fresh weight of the sample at ith given time. Then, an exponential growth model (single, two parameters) was calculated for each replicate (adjusted *R*^2^>0.96) using the software SigmaPlot (SigmaPlot, version 12.0, Systat Software Inc., Chicago, IL, USA) (Supplementary Fig. S1A, B). Subsequently, Ψ isotherms were elaborated as follows. First, for each replicate, Ψ values were converted to 1/Ψ and the linear relationship between 1/Ψ and WL after turgor loss was calculated with a linear model using the last five points of the isotherms (i.e. 1/Ψ values corresponding to the highest WL values). This linear model represents the relationship between 1/osmotic potential (π) and WL (Supplementary Fig. S1C, D). Then, to derive π, a controlled dehydration at different WL steps was generated using the slope and intercept coefficients of the 1/π versus WL relationship. Secondly, for each WL step, the exponential growth model previously calculated was used to estimate corresponding Ψ values, and the turgor pressure (P_T_) values were obtained as:


$$\begin{array}{l} P_{T}=-\Pi +\Psi \end{array}$$

These steps were repeated until P_T_ was equal to zero and the corresponding Ψ was considered as Ψ_tlp_ (Tyree and Hammel, 1972). RWC_tlp_ was calculated as follows:


$$\begin{array}{l} RWC_{tlp}=(FW_{tlp}-DW)/DW\times 100 \end{array}$$

where FW_tlp_ is the fresh weight at the turgor loss point and DW is the dry weight. FW_tlp_ was obtained as follows:


$$\begin{array}{l} FW_{tlp}=IFW-WL_{tlp} \end{array}$$

where WL_tlp_ is the water at the turgor loss point.

### Measurements of acoustic emissions

On measurement day, eight samples of approximately the same size and weight (~96.7 ± 5.5 mg) for each species and each health status (i.e. living and devitalized thalli of *F. caperata* and *L. pulmonaria*) were selected, rehydrated one last time as described above, and blotted with adsorbent paper. Four samples of each species and each health status were selected to measure the UAEs. Approximately 360 mg of Anagel™ Ultrasound gel (Ana Wiz Ltd, Chiltern Works, Chiltern Drive, Surbiton, UK) were spread over the samples’ surface where the UAE sensors had to be placed to improve the acoustic coupling. The FW of samples without and with Anagel™ was measured using an analytical balance (model CP64, Sartorius AG, Göttingen, Germany). UAEs were monitored with a MICRO-II EXPRESS system and 150 kHz resonance sensors (R15α) connected to 2/4/6 pre-amplifiers set to 40 dB (Physical Acoustics Corp., Wolfegg, Germany). The threshold was set to 30 dB, and peak definition time, hit definition time, and hit lockout time were set to 200, 400, and 2 μs, respectively. Recording of UAEs was performed with AEwin software (Mistras Holdings Corp., Princeton, NJ, USA) through which time, number, amplitude, and absolute energy of UAEs were registered. Weights of ~88 g were placed on the resonance sensors to prevent them moving during sample desiccation (Supplementary Fig. S2). The remaining four samples were prepared in the same way to reproduce the recording conditions, but the sensors were not attached to the AE system. In this way, for each sample whose acoustic emissions were continuously monitored, a paired sample was used to record FW every 30 min. Samples were left dehydrating at a room temperature of 24 °C and relative humidity of 20%. In order to couple UAEs with FW, the number of UAEs of each sample was noted every time FW was measured. Once UAEs reached a stationary phase and FW did not change any further, samples were placed in an oven for 24 h at 80 °C and DW was measured. UAEs were also recorded on paper samples (grade 2 qualitative filter papers, Whatman; Supplementary Fig. S3) of similar size to the lichen samples as a control for UAEs from the environment and/or the desiccating Anagel™ Ultrasound gel. To check for the lack of background noise recorded by the instruments, the sensors were attached directly to the worktable before the experiments began, and UAEs were monitored for ~24 h (Supplementary Fig. S3). After the recording, all raw UAE data were filtered by removing acoustic emissions that occurred in <1 s after the previous one, as they potentially are background noise. The relative number of cumulative UAEs (rcUAEs) of each sample was calculated as follows:


$$\begin{array}{l} rcUAE=\frac{UAE_{i}}{UAE_{\max}}\times 100 \end{array}$$

where UAE_i_ is the number of the cumulative UAEs at the ith given time and UAE_max_ is the maximum number of cumulative UAEs recorded on that sample. UAEs were always expressed as a function of RWC because it was impossible to record UAE and Ψ simultaneously due to the time needed to measure a stable Ψ with WP4C.

### Low-temperature scanning electron microscopy

To verify if UAEs originated from structural modifications of the thalli occurring during desiccation, LTSEM observations were carried out.

Samples of both species were rehydrated to full turgor as previously mentioned, then three sets of 3–4 samples were formed: fully turgid samples and samples equilibrated at 20 °C in a growth chamber over a saturated NaCl solution (relative humidity 75%) and over silica gel (~3% relative humidity) to obtain RWCs of 16% and 5%, respectively, namely above and below the RWC at which the UAE occurred (RWC_UAE_) (see the Results). To verify when the RWC of samples was at equilibrium with the relative humidity, their FWs were measured daily and their RWC was calculated as follows:


$$\begin{array}{l} RWC=(FW-DW)/DW\times 100 \end{array}$$

For the analysis, lobe pieces of ~2 × 6 mm were cut, one per sample, at 2 mm from the sample margin and attached to a cryo-holder using colloidal graphite. Four pieces from different treatments were mounted in each cryo-holder. Cryo-holders were then plunge-frozen in LN_2_ slush and transferred with a transfer rod module into the cryo-preparation system (PP3010T, Quorum Technologies, Laughton, Sussex, UK). Sample manipulation before freezing was maintained below 5 min to avoid sample rehydration. Samples were mechanically freeze-fractured and then freeze-etched by sublimation for 5 min at –90 °C. A thin layer of platinum was sputtered onto the specimens for 90 s with a current of 10 mA and afterward transferred into a field emission scanning electron microscope (SCIOS 2 FESEM microscope, FEI Company Ltd, Hillsboro, OR, USA). Images were recorded at an accelerating voltage of 3 kV using the in-lens back-scattered electron detector. The images are photographic negatives; hence, protuberant elements of the fractured/etched surface are most heavily coated with platinum and appear white/bright.

### Statistical analyses

Statistically significant differences of Ψ_tlp_ and RWC_tlp_ between the two species were evaluated through Student’s *t*-test and using the function t.test of R (R, version 4.1.3, R Foundation; R Core Team, 2022) package ‘stats’. Normality and homoscedasticity assumptions were tested using the Shapiro–Wilk test of normality (function shapiro.test of the R package ‘stats’) and an *F*-test to compare the variances of each parameter for the two species (functions ‘var.test’ of the R package ‘stats’).

The relationship between *F*_v_/*F*_m_ (response variable) and Ψ (predictive variable) was tested by fitting a sigmoidal model using the fitcond function in the ‘fitplc’ R package (Duursma and Choat, 2017). To assess Ψ values inducing a significant drop of *F*_v_/*F*_m_, the breakpoint and the associated confidence intervals (CIs) of the *F*_v_/*F*_m_–Ψ relationship in each species were calculated using the functions segmented and confint.segmented in the ‘segmented’ R package (Muggeo, 2017).

The relationship between RWC and relative UAE was evaluated using a linear mixed-model fitted using function lme of the R package ‘nlme’ (Pinheiro *et al.*, 2023). RWC was set as the explanatory variable, while UAE was the response variable, and the sample (ID) was the random effect. As described above, the breakpoint and its associated CIs of the UAE–RWC relationship were estimated to assess RWC values corresponding to an increase in acoustic emissions (RWC_UAE_).

A third-grade polynomial regression model was used to describe the relationship between absolute energy and amplitude of living and devitalized samples of both species, setting amplitude as the explanatory variable and the absolute energy as the response variable.

## Results

### Water potential and relative water content of lichen thalli

**Fig. 1.**
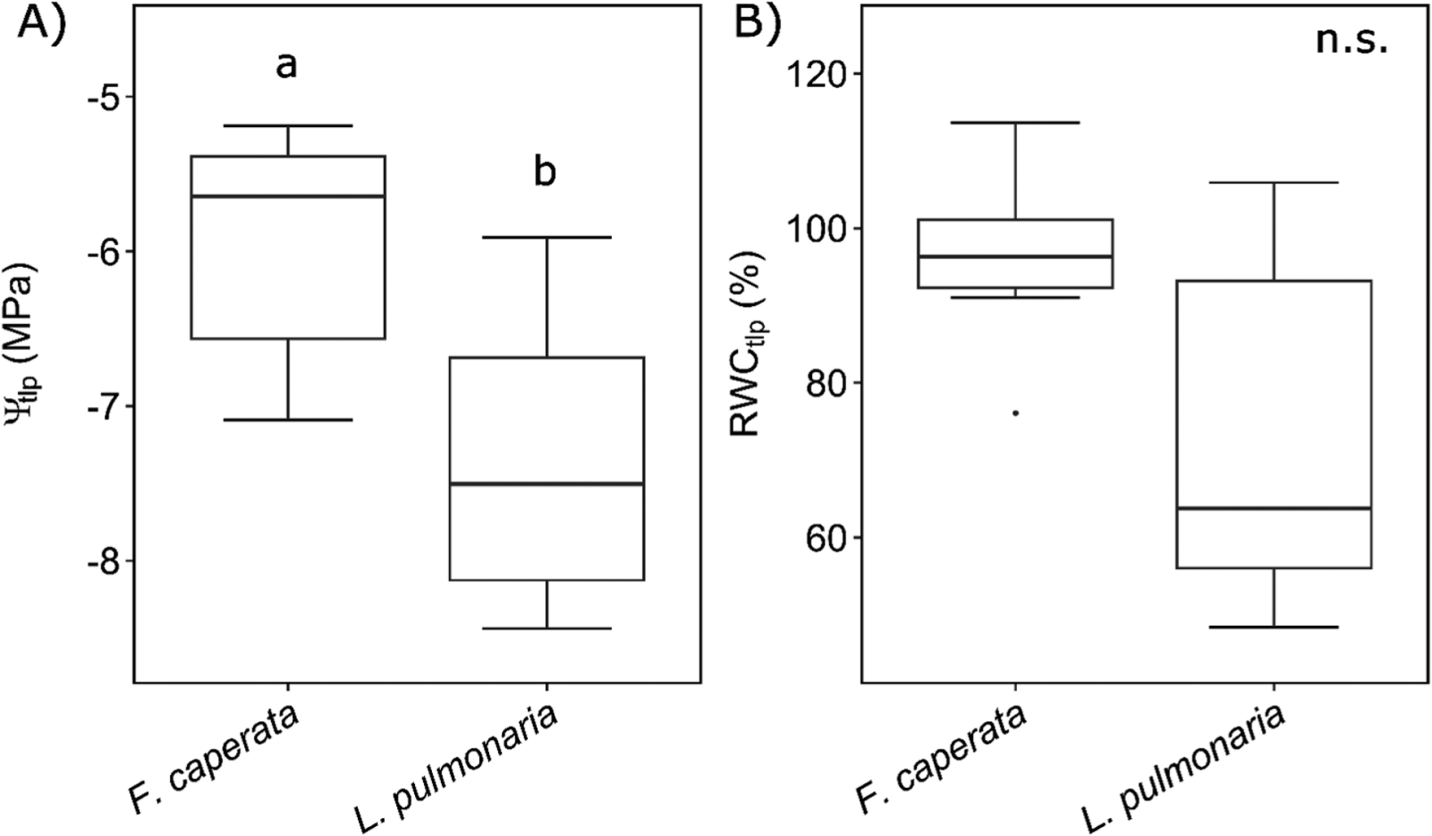
Water potential and relative water content at the turgor loss point. Boxplots (*n*=6) of water potential at the turgor loss point (Ψ_tlp_, A) and relative water content at the turgor loss point (RWC_tlp_, B) in *Flavoparmelia caperata* and *Lobaria pulmonaria*. Whiskers represent values lower or higher than the 25th and 75th percentile ±1.5×interquartile range, respectively. Different letters indicate statistically significant differences (*t*-test, *P*-value <0.05).

Water potential at turgor loss point (Ψ_tlp_) was significantly higher in *F. caperata* than in *L. pulmonaria* (–6.0 ± 0.8 MPa and –7.4 ± 1.0 MPa, respectively) (Fig. 1), while no statistically significant differences between the two species were found for relative water content at turgor loss point (RWC_tlp_), being 96 ± 12% for *F. caperata* and 73 ± 25% for *L. pulmonaria* (Supplementary Table S1).

### PSII efficiency in relation to water potential

**Fig. 2.**
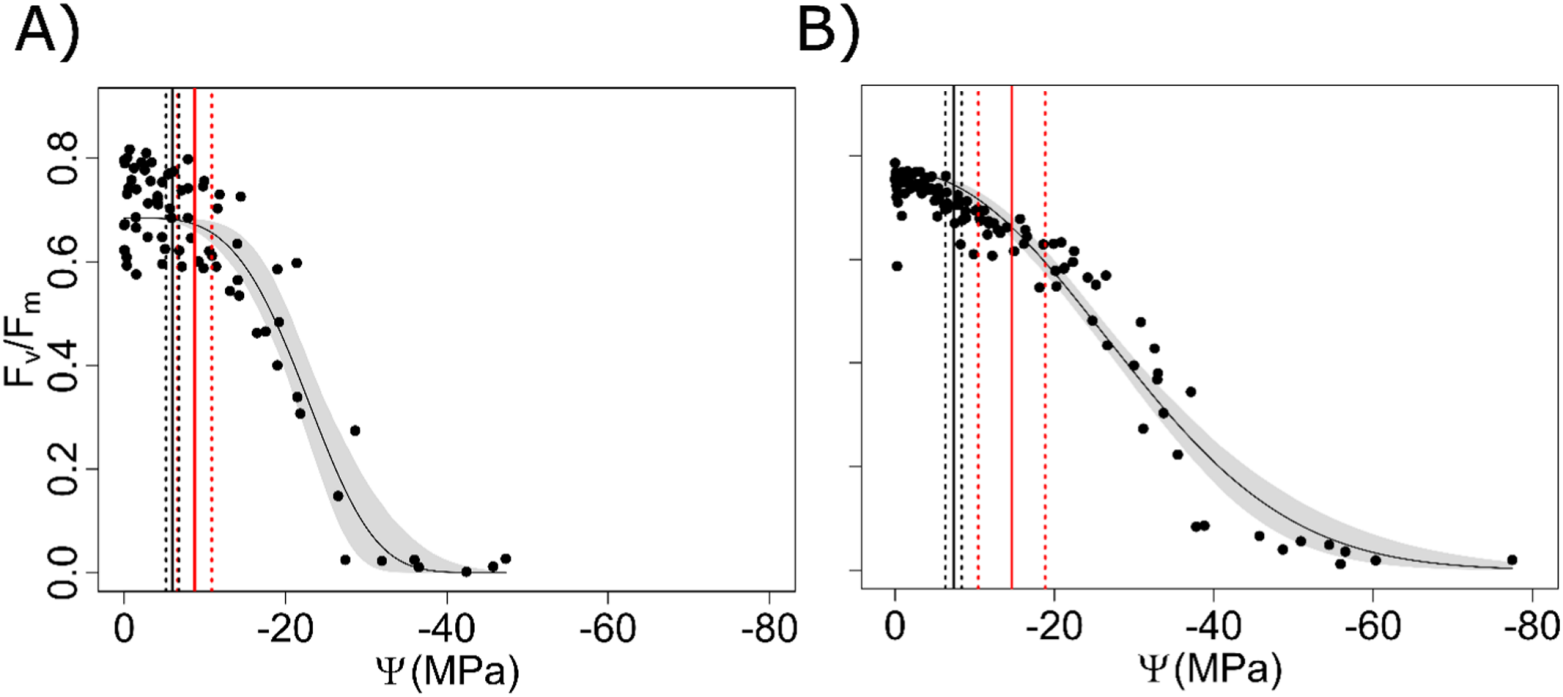
Relationship between PSII maximum quantum yield and water potential. The relationship between PSII maximum quantum yield (*F*_v_/*F*_m_) and water potential (Ψ) in *Flavoparmelia caperata* (A) and *Lobaria pulmonaria* (B). Grey bands represent the confidence interval of the regression model. Solid and dotted red lines correspond to the breakpoint of the regression models and their confidence interval, respectively. Solid and dotted black lines represent the mean value of Ψ at turgor loss point (Ψ_tlp_) and the SD (*n*=6), respectively.

During desiccation, PSII maximum quantum yield (*F*_v_/*F*_m_) progressively decreased from values of 0.680 ± 0.079 and 0.742 ± 0.046 at full turgor (Ψ=0 MPa) to 0 at Ψ values equal to –38.2 ± 7.3 MPa (RWC=15 ± 2%) and –52.7 ± 11.5 MPa (RWC=18 ± 3%) in *F. caperata* and *L. pulmonaria*, respectively. In *F. caperata*, *F*_v_/*F*_m_ started to decrease significantly at –8.7 MPa (calculated breaking point; RWC=87 ± 12%), with a CI ranging from –6.6 MPa to –10.8 MPa. In *L. pulmonaria*, *F*_v_/*F*_m_ started to decrease significantly at –14.6 MPa (RWC=51 ± 18%; CI –10.4 MPa to –18.9 MPa). Hence, in both species, *F*_v_/*F*_m_ started to significantly decrease at Ψ values slightly lower than Ψ_tlp_ (Fig. 2).

### Ultrasonic acoustic emissions during desiccation

**Fig. 3.**
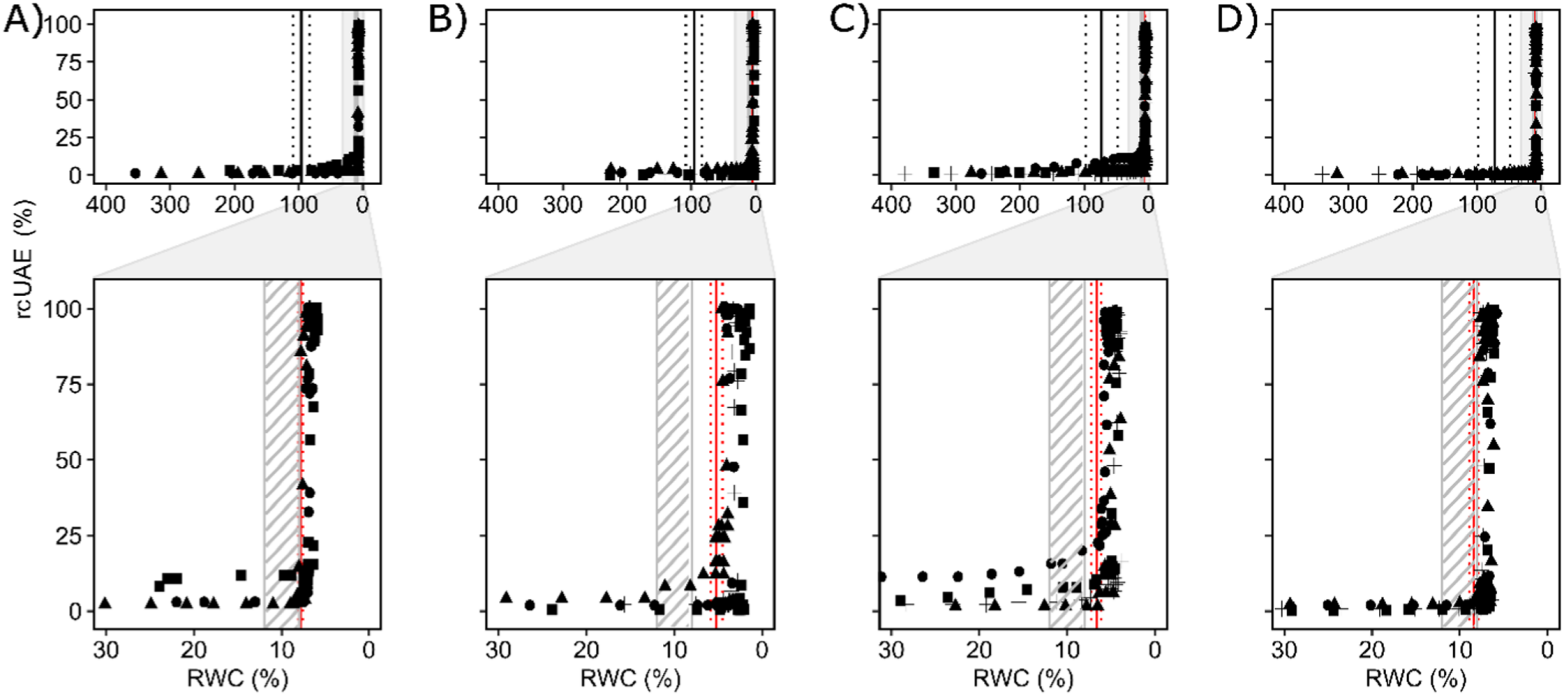
Relationships between relative cumulative ultrasonic acoustic emissions and relative water content. The relationships between relative cumulative ultrasonic acoustic emissions (rcUAE, %) and relative water content (RWC, %) on living (A, C) and devitalized (B, D) samples of the lichens *Flavoparmelia caperata* (A, B) and *Lobaria pulmonaria* (C, D), respectively, shown for the whole spectrum of observed RWC (upper panel row) and for the 30–0 RWC range (lower panel row). Solid and dotted black lines represent the mean value of RWC at turgor loss point (RWC_tlp_) and the SD (*n*=6), respectively. The shaded areas with 45° grey lines represent the RWC range at which cells make the transition to a glassy state according to Candotto Carniel *et al.* (2021). Solid and dotted red lines represent the RWC values at which relative UAE significantly (Supplementary Table S3) increased (RWC_UAE_) and the lower and upper limits of their confidence intervals. Squares, triangles, circles, and crosses represent different samples.

UAEs were almost absent in living and devitalized samples (Supplementary Table S2) of both species at an RWC between 380% and 30%, with relative cumulative UAE (rcUAE) ranging from 0% to 11%. rcUAE produced by *F. caperata* during desiccation strongly increased between 8% and 7% of RWC in living samples and between 6% and 5% of RWC in devitalized samples (Fig. 3A, B). Similarly, in living and devitalized samples of *L. pulmonaria*, rcUAE strongly increased between 7% and 6% of RWC and between 9% and 8% of RWC, respectively (Fig. 3C, D). RWC values corresponding to a significant increase of rcUAE (RWC_UAE_) were statistically different between the living and devitalized samples in both species (Supplementary Table S3).

**Fig. 4.**
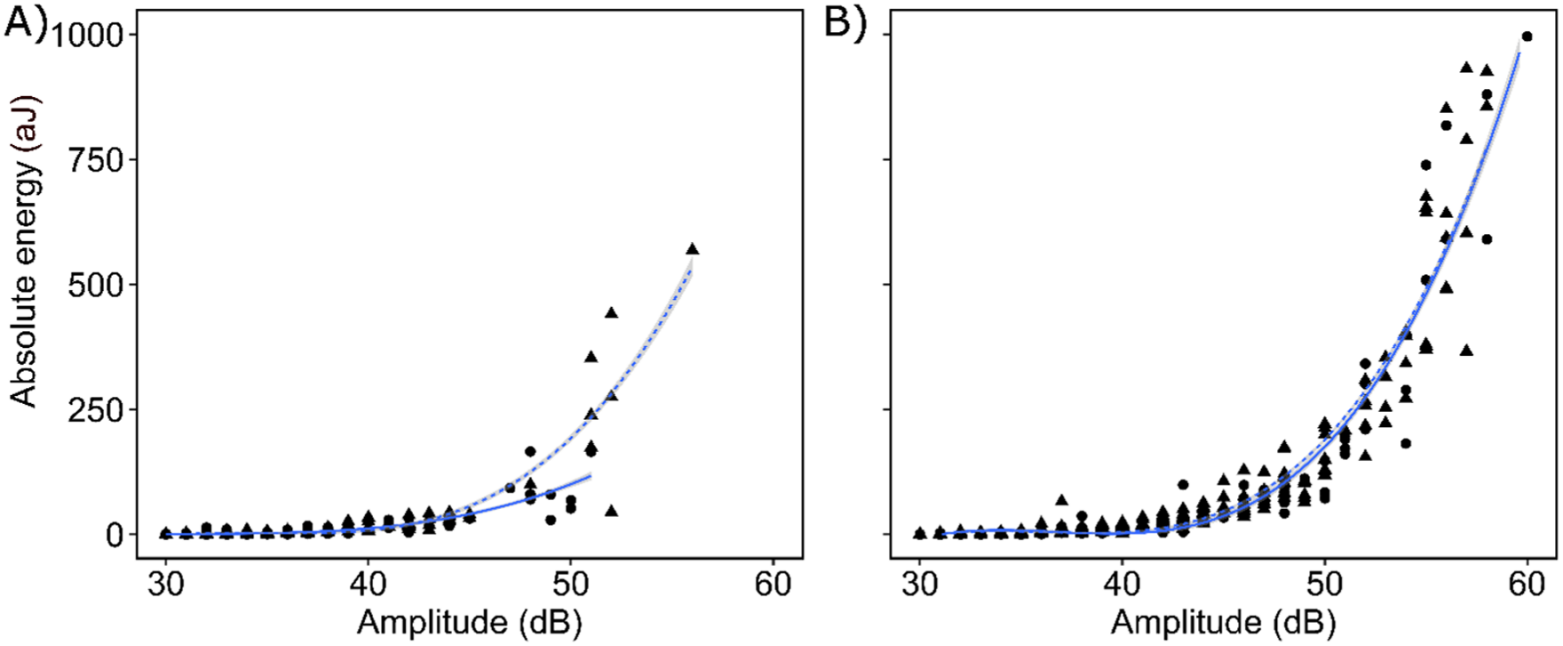
Relationship between amplitude and absolute energy of ultrasonic acoustic emissions. The relationship between amplitude (dB) and absolute energy (aJ) of ultrasonic acoustic emissions (UAEs) recorded on living (circles) and devitalized (triangles) samples of the lichens *Flavoparmelia caperata* (A) and *Lobaria pulmonaria* (B). Solid and dashed blue lines indicate third-grade polynomial regression models for living and devitalized samples, respectively, while grey areas indicate their confidence intervals.

**Fig. 5.**
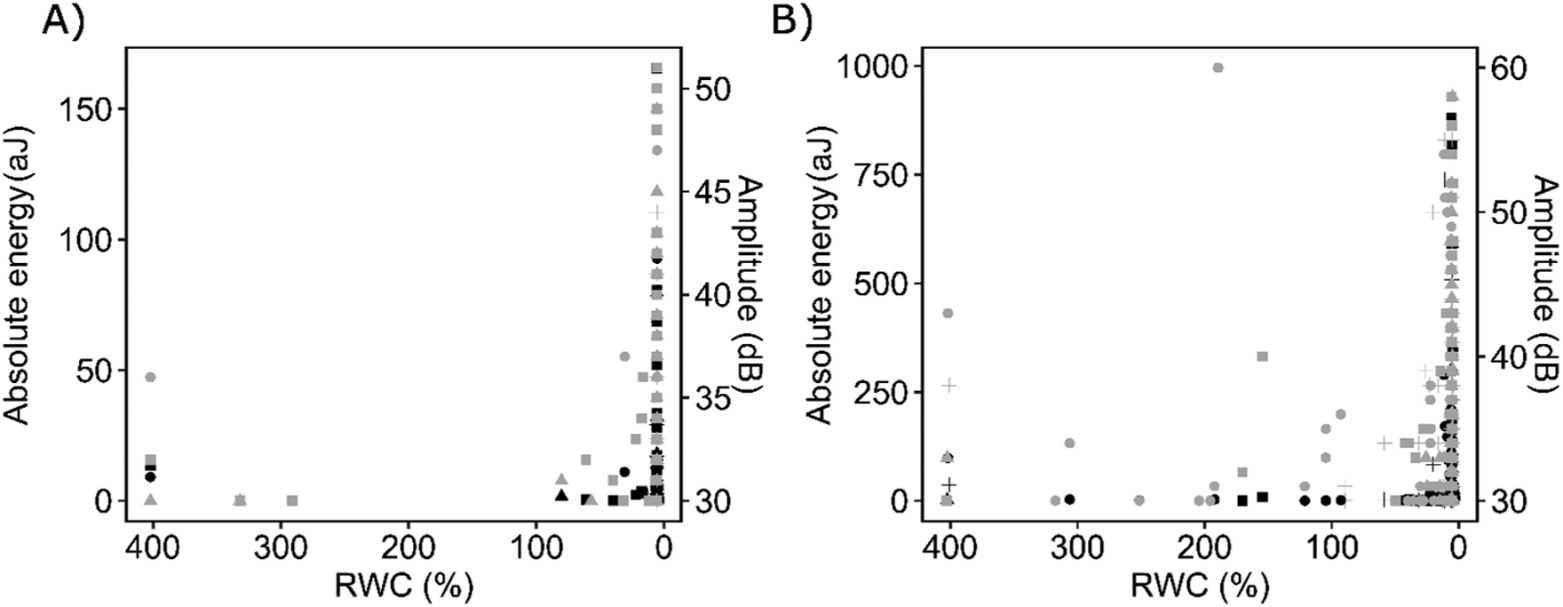
Relationship between relative water content, amplitude, and absolute energy. The relationship between relative water content (RWC, %), amplitude (dB, right *y*-axis), and absolute energy (aJ, left *y*-axis) on living samples of the lichens *Flavoparmelia caperata* (A) and *Lobaria pulmonaria* (B). Black dots indicate absolute energy values, while grey dots indicate amplitude values. Different shapes of the dots represent different samples.

The relationship between amplitude and absolute energy of UAE followed a slightly different trend in living and devitalized samples of *F. caperata*, while it was almost identical in *L. pulmonaria* (Fig. 4). In both species, the UAEs at low RWC are characterized by higher amplitude and absolute energy than those occurring at high RWC (Fig. 5). Moreover, UAE in *F. caperata* living samples corresponded to lower absolute energy and amplitude values than those in *L. pulmonaria.* The same pattern was also observed for devitalized samples of both species (Supplementary Fig. S4).

### LTSEM observations

**Fig. 6.**
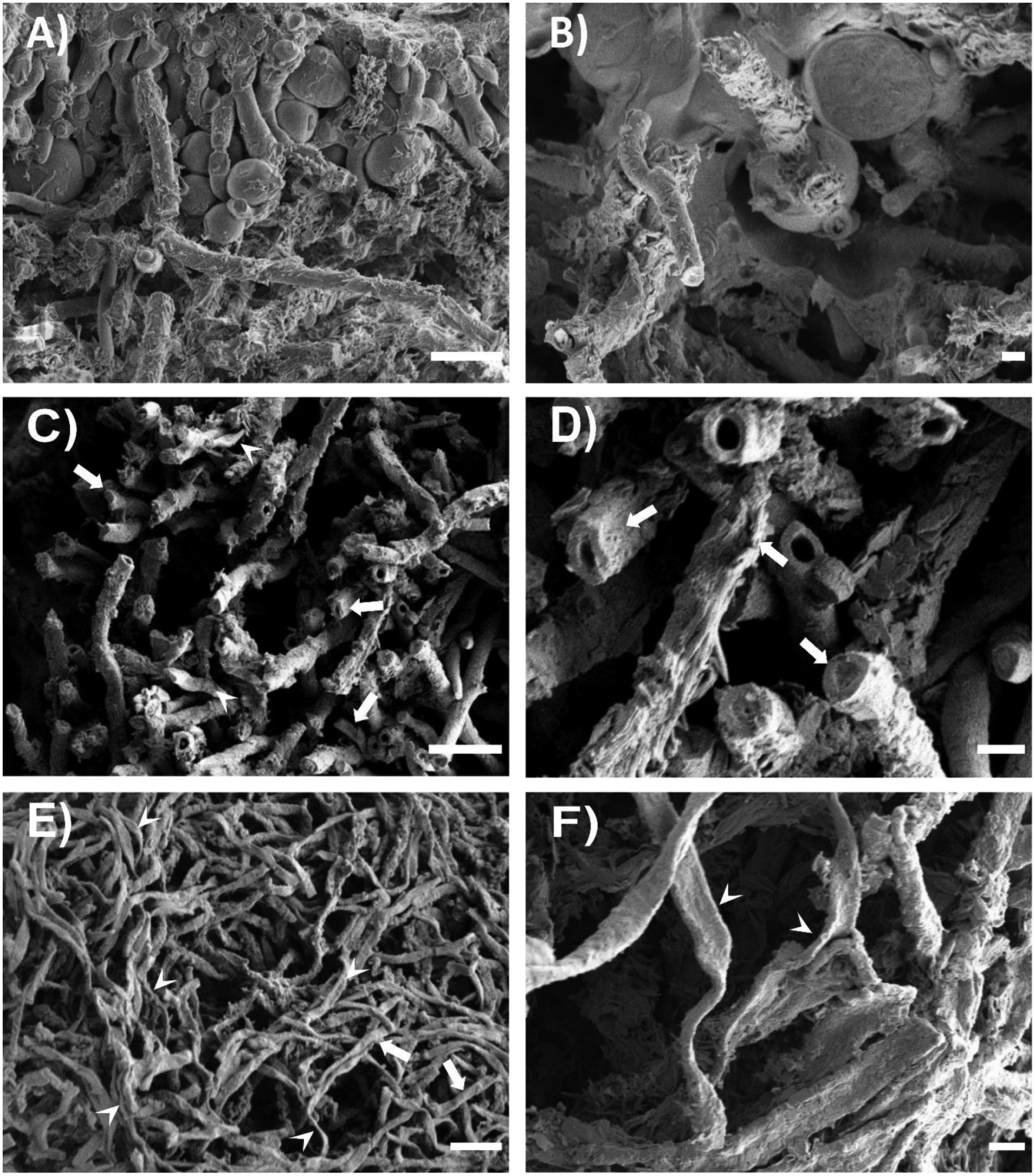
LTSEM micrographs of *Flavoparmelia caperata*. Micrographs of cryo-fractured *Flavoparmelia caperata* at different water contents, namely 167.1 ± 18.3% RWC (A, B), 16.7 ± 1.1% RWC (C, D), and 5.1 ± 0.9% RWC (E, F): overview micrographs (A, C, E) and detail of each condition (B, D, F). White arrows indicate slightly shrunken hyphae; white arrowheads indicate completely shrunken hyphae. Scale bars A, C, E=10 μm; B, D, F=2 μm.

**Fig. 7.**
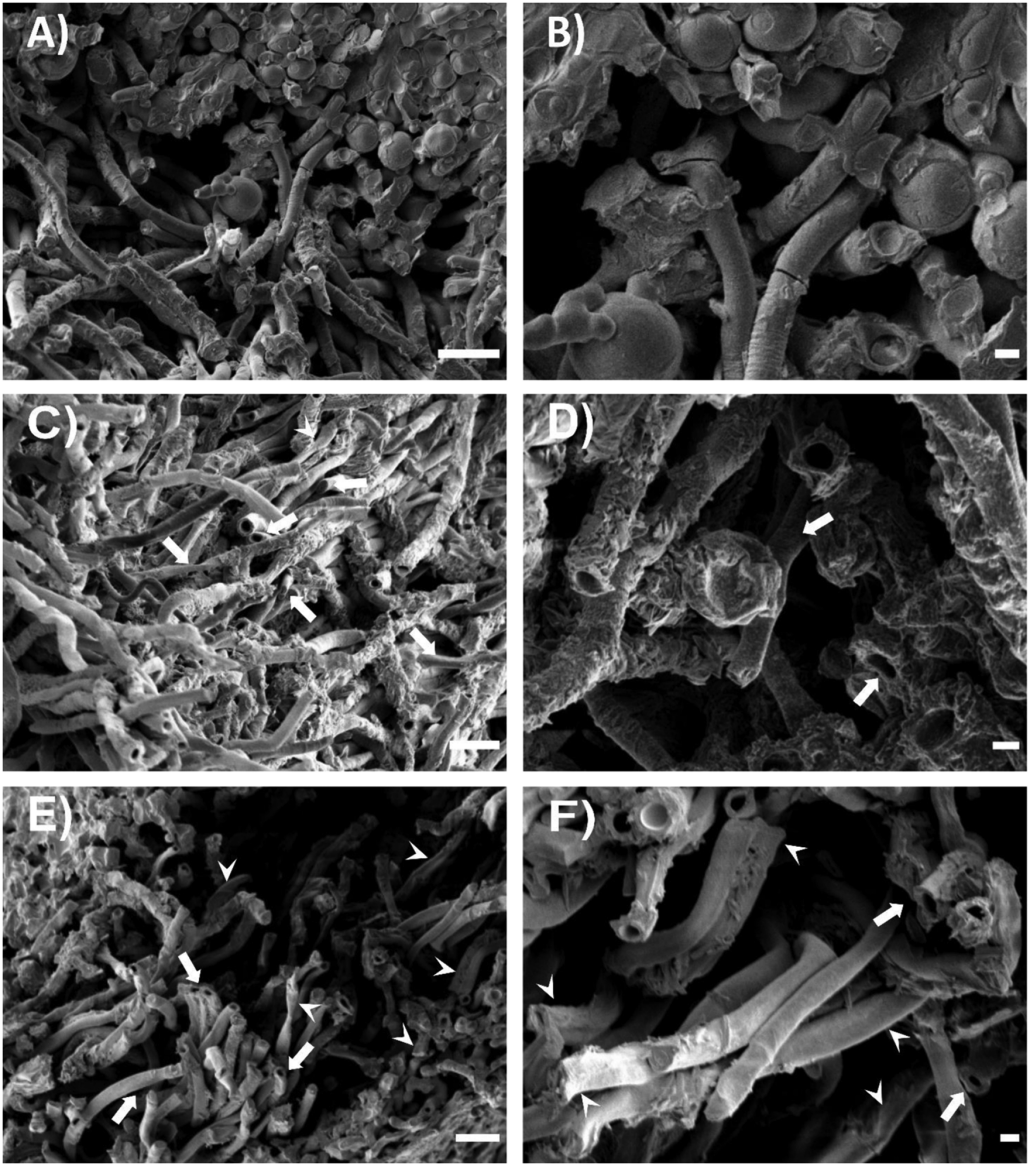
LTSEM micrographs of *Lobaria pulmonaria*. Micrographs of cryo-fractured *Lobaria pulmonaria* lobes at different water contents, namely 151.4 ± 4.1% RWC (A, B), 16.4 ± 1.4% RWC (C, D), and 5.3 ± 0.5% RWC (E, F): overview micrographs (A, C, E) and detail of each condition (B, D, F). White arrows indicate slightly shrunken hyphae; white arrowheads indicate completely shrunken hyphae. Scale bars A, C, E=10 μm; B, D, F=2 μm.

*Flavoparmelia caperata* and *L. pulmonaria* at full turgor had an RWC of 167 ± 18% and 151 ± 4%, respectively (Supplementary Table S4). At this water content, no cavities were observed in cross-views of cortical and medullary hyphae (Figs 6A, B, 7A, B; Supplementary Figs S5A, B, S6A, B). Samples of both species showed spherical photobionts and fungal hyphae of cylindrical shape with a visible cell content where the cryo-fracture was carried out (Figs 6A, B, 7A, B). The medullary hyphae in *F. caperata* were heavily encrusted by crystals of lichen substances and calcium oxalates, more than in *L. pulmonaria*, which masked their morphology (Fig. 6A–C). Samples equilibrated over NaCl-saturated solution had an RWC of 17 ± 1% (*F. caperata*) and 16 ± 1% (*L. pulmonaria*)— above the RWC_UAE_ (Supplementary Table S4). At these RWCs, cryo-fractured cortical and medullary hyphae of both species had cavities inside the protoplast when observed in cross-view, sometimes occupying almost the whole cell volume (Figs 6C, 7C), with others appearing only as small niches (Supplementary Fig. S7). Moreover, samples of both species showed partially shrunken photobiont cells (Fig. 7D; Supplementary Figs S5C, S6C). Jointly, a few cortical hyphae were slightly collapsed (Supplementary Figs S5C, D, S6C, D) and a high number of medullary hyphae were partially shrunken (Figs 6C, D, 7C, D). Samples equilibrated over silica had an RWC of 5 ± 1% and 5 ± 0.5% for *F. caperata* and *L. pulmonaria*, respectively—below the RWC_UAE_ (Supplementary Table S4). In both species, cortical and medullary hyphae had cavities in the protoplast (Fig. 7E, F; Supplementary Figs S5E, F, S6E, F), more frequently in the medullary hyphae. All photobiont cells were completely shrunken (Fig. 7E; Supplementary Figs S5E, S6E). Some degree of cell shrinking was also observed in cortical hyphae, which showed a less round shape with respect to full turgor (Supplementary Figs S5E, F, S6E, F), while almost all medullary hyphae were heavily shrunken, with flattened portions visible along the hyphae length (Figs 6E, F, 7E, F).

## Discussion

In lichens, the relationship between water status and CO_2_ gas exchange has been extensively studied (Bilger *et al.*, 1989; Lange *et al*., 2001; Gasulla *et al.*, 2021). Previous studies on foliose or umbilicate thalli reported horizontal differences in the deactivation and reactivation kinetics of photosystems during desiccation. This pattern is related to horizontal intrathalline differences in water content, as marginal parts of thalli tend to lose water faster than central parts (e.g. Schroeter *et al.*, 1992; Phinney *et al.*, 2018; Solhaug *et al.*, 2021). However, recent comparative analyses suggested a vertical variability in water potential (Ψ) within the thallus, namely from the lower to the upper cortex, similarly to the horizontal one (Kosugi *et al.*, 2009; Petruzzellis *et al.*, 2018). Specifically, it has been hypothesized that the hydration status of the two symbionts within the thallus is uncoupled during desiccation, possibly as a result of cavitation events in fungal hyphae, observed by Scheidegger *et al.* (1995) and Honegger (e.g. Honegger, 1996, 1998) as gas bubbles in the protoplast of hyphae. The expansion of a gas bubble could cause water movements towards the photobiont layer, delaying its desiccation and maintaining a higher Ψ than in the mycobiont layers. We tested this hypothesis by (i) verifying whether there is a significant decrease in PSII maximum quantum yield (*F*_v_/*F*_m_) at Ψ values more negative than Ψ at turgor loss point (Ψ_tlp_) in *F. caperata* and *L. pulmonaria*; (ii) characterizing UAEs to verify whether they could be associated with cavitation events; (iii) verifying whether UAEs occurred at water contents compatible with cavitation-induced water movement in the thalli, specifically at RWC values close to the turgor loss point (RWC_tlp_); and (iv) performing a morphological characterization of the thalli during desiccation using LTSEM.

### Ψ–*F*_v_/*F*_m_ relationship

Since Ψ_tlp_ has been proposed to play a crucial role in triggering a significant decrease in *F*_v_/*F*_m_ in both *F. caperata* and isolated *T. gelatinosa* (Petruzzellis *et al.*, 2018), we aimed at testing whether this phenomenon also occurred in our samples. Our findings are consistent with those of Petruzzellis *et al.* (2018) for *F. caperata*, who reported a similar Ψ_tlp_ value, while to our knowledge this is the first time that Ψ_tlp_ of *L. pulmonaria* is reported. Ψ_tlp_ was significantly different between the two species, while there were no significant differences in RWC_tlp_ (Fig. 1), due to the high variability in *L. pulmonaria*. Nardini *et al.* (2013) analysed water relation parameters in six *Peltigera* species and found that species adapted to more xeric environments had lower Ψ_tlp_ than those adapted to more mesic environments. Moreover, they showed that lichens with thick fungal cell walls had a high modulus of elasticity (i.e. stiff cell walls), which was negatively correlated with Ψ_tlp_. Therefore, the difference in Ψ_tlp_ between *F. caperata* and *L. pulmonaria* might depend mainly on the cell wall composition of the two species and not on adaptations to environments with different water availability, as instead demonstrated for congeneric species (Nardini *et al.*, 2013).

Measuring *F*_v_/*F*_m_ until complete inactivation of PSII provided useful information on the water status of the photobiont inside the thallus during desiccation. In *F. caperata*, *F*_v_/*F*_m_ reached values equal to 0 at Ψ values of –38.18 ± 7.30 MPa, and in *L. pulmonaria* at Ψ values of –52.67 ± 11.46 MPa (Fig. 2). The sigmoidal shape of the curves and the Ψ values at which *F*_v_/*F*_m_ equals 0 are in good agreement with previous findings on *Xanthoria elegans* (Barták *et al.*, 2005) and on *Umbilicaria arctica* and *U. hyperborea* (Barták *et al.*, 2015). We recorded a significant decrease in *F*_v_/*F*_m_ at Ψ values slightly more negative than Ψ_tlp_ in both species (Fig. 2), consistent with findings by Petruzzellis *et al.* (2018). Assuming that *F*_v_/*F*_m_ can be used as a proxy of photobiont Ψ within the thallus, our results suggest that the Ψ drop in the photobiont was delayed compared with the thallus and thus the photobiont reached the Ψ_tlp_ after that of the surrounding fungal pseudo-tissues. This suggests that processes related to the loss of turgor in mycobiont hyphae might play a role in maintaining a higher Ψ at the photobiont level. The hyphal cavitation hypothesis by Scheidegger *et al.* (1995) would justify an additional water supply to the photobiont cells, which would delay the drop of *F*_v_/*F*_m_. This hypothesis might be plausible especially considering that, in other organisms, cavitation events occur at Ψ values ranging roughly from close to 0 to –10 MPa (Milburn, 1970; Dixon *et al.*, 1984; Tyree and Dixon, 1986; Brodribb *et al.*, 2020; Sorek *et al.*, 2021)—close to the Ψ at which *F*_v_/*F*_m_ starts to decrease in lichens (Petruzzellis *et al.*, 2018).

### UAE signals and their characterization

Cavitation events can occur in vascular plants (Lo Gullo and Salleo, 1992; Salleo *et al.*, 2000; Mayr *et al.*, 2007) as well as in other organisms such as ferns (Ritman and Milburn, 1990), fungal ascospores (Ingold, 1956; Milburn, 1970), and mosses (Brodribb *et al.*, 2020). In vascular plants, though, cavitation by heterogeneous nucleation (i.e. air-seeding mechanism; Tyree and Zimmermann, 2002) occurs in the xylem conduits (i.e. structures made of cell walls of dead cells), and the phenomenon is quite intense, as air can fill a conduit in an explosive way (Zimmermann, 1983). On the other hand, the purported cavitation observed in ascospores occurred without damaging the cell irremediably, as the ascospores were still able to germinate after the appearance (during desiccation) and the disappearance (during rehydration) of the resulting gas bubbles (Ingold, 1956), making a cavitated hypha a possibility.

In vascular plants, the energy released by cavitation events is accompanied by a shock wave detectable as ultrasonic emission. Therefore, if the hypothesis of hyphal cavitation pushing water towards the photobiont layer were to be correct, the UAE peak should have occurred at RWC values between RWC_tlp_ and those corresponding to the start of the *F*_v_/*F*_m_ decrease. However, none of the very few UAEs measured before complete desiccation occurred at RWCs in that interval, greatly weakening the viability of the hyphal cavitation hypothesis. The few rcUAE (0% to 11% maximum) recorded between 380% and 30% of RWC are ascribable to the background noise recorded in some cases when measuring UAEs on paper and on the table alone (Supplementary Fig. S3E, F). Therefore, alternative mechanisms for the prolonged hydration of the photobiont should be explored. One possibility is the accumulation of solutes in extracellular spaces near the photobiont or within the photobiont cells (Honegger, 1997; Petruzzellis *et al.*, 2018). This accumulation could lead to a decrease in osmotic potential, triggering passive movements of water towards the photobiont.

Interestingly, most UAEs recorded for both species occurred at an RWC much lower than the respective RWC_tlp_; that is, between 8.4% and 5.2% of RWC on a dry mass basis (Fig. 3; Supplementary Tables S1, S3). At this RWC, both mycobiont and photobiont cells are very likely to be in a glassy state (Buitink and Leprince, 2008; Verhoeven *et al.*, 2018), as the transition from a rubbery to a glassy state occurs between 12% and 8% RWC on a dry weight basis (at 20 °C) (Candotto Carniel *et al.*, 2021). Below this RWC range, the residual intermolecular water is so scarce that molecular mobility is reduced (Candotto Carniel *et al.*, 2021) and there is no free water left to be pushed in any direction. In addition, the UAE signals were similarly recorded in both living and devitalized samples of both species (Fig. 3), indicating that the energy-releasing phenomena triggering UAEs are not likely to be related to the metabolic activity or vitality of the samples. Consequently, a possible source of UAEs could be the morphological and/or structural changes of the cell walls.

UAEs in vascular plants can have various sources, for example by vibrating cell walls of xylematic conduits after the release of tensions due to cavitation events (Zimmermann, 1983; Johnson *et al.*, 2009, 2012), by tensional shrinkage of the cell walls that can lead to cracks and micro-fractures (Cunderlik *et al.*, 1996; Kowalski and Smoczkiewicz, 2004; Rosner *et al.*, 2009), by oscillation of hydrogen bonds in water after pressure release, or by state transition from liquid water to vapour (for a review, see De Roo *et al*., 2016). As a consequence, determining the origin of bulk UAE data is not straightforward and requires the analysis of other descriptors, such as the amplitude (dB) and the absolute energy (aJ) (Mayr and Rosner, 2011). The ascending pattern of absolute energy in relation to the amplitude of the UAE detected in this study was similar in living and devitalized samples of both species (Fig. 4), suggesting a common origin (Kim *et al.*, 2005; Wolkerstorfer *et al.*, 2012). Furthermore, these patterns, where energy increases without reaching a descending phase, are more similar to those detected in drying wood, whose origin was related to the increase of tension on wood fibres at low water content, leading to cracks and fractures occurring at the level of the cell walls (Cunderlik *et al.*, 1996; Kowalski and Smoczkiewicz, 2004). In our samples, as well as in drying wood, as RWC decreased, the absolute energy and amplitude of UAE increased, in both living (Fig. 5) and devitalized samples (Supplementary Fig. S4). Therefore, considering the low RWC at which they occur and the glassy state of cells (i.e. low molecular mobility), we propose that the cause of UAEs detected here is structural changes inside thalli, most probably due the tension exerted on hyphal cell walls and suddenly released upon desiccation (e.g. shear forces due to shifts between adjacent cells or stretching and compressing of cells).

### Morphological characterization of thalli during desiccation

To investigate the occurrence of possible structural changes within the thalli that could justify the detected UAEs, we performed LTSEM observations of different hydration treatments in both species. LTSEM observations can give very important information on the sample conditions at a given RWC although it is not a quantitative method. Samples at full turgor (Figs 6A, B, 7A, B) showed very similar conditions to those already presented in previous LTSEM (Honegger, 1995; Scheidegger *et al.*, 1995; Honegger *et al.*, 1996; Kappen *et al.*, 1996) and quick-freeze deep-etch electron microscopy (Goodenough *et al.*, 2021; Roth and Goodenough, 2021) studies on different lichen species, with no free water inside the thallus. Comparing these observations with those conducted on samples at very low RWCs, above and below RWC_UAE_, we noted an increase in cavities within the hyphal protoplast in both species, (Figs 6C, D, 7C–F). Analogous cavities, initially interpreted as protoplast shrinkage (Brown *et al.*, 1987), were later interpreted as ‘gas bubbles’ of unknown origin (Scheidegger, 1994; Honegger, 1995, 1997; Scheidegger *et al.*, 1995; Honegger *et al.*, 1996). They were thought to keep the hyphal cell membrane in contact with the cell wall during desiccation to avoid plasmolysis, as hyphae cell walls are relatively rigid and unable to fold, along with the significant change in volume occurring in the desiccating protoplast (Honegger, 1995). According to our observations, this hypothesis seems plausible, especially to avoid cell damage in the early stages of desiccation (i.e. from water contents <85%; Scheidegger *et al.*, 1995). However, considering that we did not detect a significant increase of UAE at the RWC in which these cavities occur, these bubbles cannot be linked to ‘explosion-like’ cavitation events (Scheidegger *et al.*, 1995). Furthermore, LTSEM observations on *Pinus nigra* needles during desiccation revealed that firstly the tracheids collapsed, then embolized, with subsequent cell wall relaxation (Cochard *et al.*, 2004). Conversely, in our samples, hyphae gradually shrank until completely flattened without cell wall relaxation, further weakening the hypothesis of hyphal cavitation. A possible origin of these gas bubbles could be like that of the concentric bodies that have been found in the hyphae of lichenized (Honegger, 2007; Goodenough and Roth, 2021) and non-lichenized fungi (Kim *et al*., 2004), as well as in other organisms of different Kingdoms (Pfeifer, 2012; Daviso *et al.*, 2013; Park and Kim, 2017). These structures have a proteinaceous wall and are filled with gas in the centre, with a diameter of ~0.3 µm in fungal hyphae (Honegger, 2007). Our findings suggest that, like concentric bodies, the observed larger cavities are filled, by diffusion, with the gases that are dissolved in the cytoplasm and are not due to cavitation events explosively occupying the protoplast.

Interestingly, in the above studies, these bubbles were always reported as fully expanded, but in our observations the cavities within the hyphae are sometimes barely noticeable (Supplementary Fig. S7). This could be due to early formation of gas bubbles or, more likely, to the observation of bubble sections with smaller diameters, for example at the poles. In any case, the fact that these small bubble fractions were surrounded by the cytoplasm allows us to confute that the observed cavities originate from protoplast shrinkage as suggested by Brown *et al.* (1987).

At RWCs above and below RWC_UAE_, we also noticed an increased number of shrunken hyphae if compared with full turgor (Figs 6, 7), with almost all hyphae shrunken in *F. caperata* and *L. pulmonaria* under the latter condition (RWC ~5%). The mycobiont hyphae (both cortical and medullary) usually do not collapse to the same extent as those of photobionts, because they have a thick and more rigid cell wall (Honegger, 1995; Honegger *et al.*, 1996). Indeed, while photobionts start to shrink already at ~95% RWC, partially shrunken hyphae were observed so far only below ~20% RWC (Scheidegger *et al.*, 1995; Honegger *et al.*, 1996). In our case, we observed a complete flattening of hyphae in both species at RWCs below RWC_UAE_. In several studies it was demonstrated that lichens have an extremely good capability to fully recover from desiccation, even at RWCs similar to those used in the present study (e.g. Kranner *et al.*, 2008; Candotto Carniel *et al.*, 2015), so hyphae are thought to be fully capable to increase turgor pressure upon rehydration also when heavily shrunken, as in our LTSEM observations (Figs 6E, F, 7E, F), without suffering irreversible damage. Considering that water contents similar to RWC_UAE_ can also be achieved in nature, the flattening of fungal hyphae could be an adaptation that allows lichens to avoid cell damage from protoplast shrinkage. LTSEM observations, together with UAE descriptors, suggest that structural changes at the hyphal level during desiccation produced the recorded UAE.

### Conclusions

The present study provides new insights into the study of water dynamics in the foliose lichens *F. caperata* and *L. pulmonaria*. Here, we tested, through the monitoring of UAE, the hypothesis that cavitation events occurring in the mycobiont cells upon turgor loss might supply additional water to the photobiont cells and thus delay the drop of *F*_v_/*F*_m_. However, we could not detect UAEs near RWC_tlp_ in either species. Therefore, our results do not corroborate the hypothesis that explosive cavitation phenomena occur when the mycobiont hyphae lose water below RWC_tlp_.

Furthermore, we recorded UAEs at very low water contents (i.e. RWC_UAE_) in both species, which led us to hypothesize, based on the analysis of amplitude and absolute energy in living and devitalized samples, that UAEs originated from strong tension exerted on hyphal cell walls. We further verified this hypothesis by LTSEM observations. At RWCs lower than RWC_UAE_, a higher number of hyphae were heavily shrunken compared with full turgor and there was no evidence of visible damage. Consequently, we propose structural changes inside thalli, most probably due to the high tension exerted on hyphae and their subsequent reversible shrinking, as the main cause of the detected UAEs.

## Supplementary data

The following supplementary data are available at *JXB* online.

Fig. S1. Typical water potential (Ψ) isotherms for the lichens *Flavoparmelia caperata* and *Lobaria pulmonaria*.

Fig. S2. The setup used for the acquisition of the ultrasonic acoustic emissions (UAEs).

Fig. S3. Relationship between time and absolute number of ultrasonic acoustic emissions (UAEs) in living and devitalized lobes of *Flavoparmelia caperata* and *Lobaria pulmonaria*, paper samples, and table.

Fig. S4. Relationship between relative water content (RWC; %), amplitude (dB), and absolute energy (aJ) on devitalized lobes of the lichens *Flavoparmelia caperata* and *Lobaria pulmonaria*.

Fig. S5. LTSEM micrographs of cryo-fractured lobes of *Flavoparmelia caperata* at different water contents.

Fig. S6. LTSEM micrographs of cryo-fractured lobes of *Lobaria pulmonaria* at different water contents.

Fig. S7. LTSEM micrographs of hyphae manifesting the onset of a ‘gas bubble’ in lobes of *Flavoparmelia caperata* and *Lobaria pulmonaria*.

Table S1. Water relation parameters derived by the elaboration of water potential (Ψ) isotherms of the lichens *Flavoparmelia caperata* and *Lobaria pulmonaria*.

Table S2. PSII maximum quantum yield (*F*_v_/*F*_m_) on living and devitalized lobes of the lichens *Flavoparmelia caperata* and *Lobaria pulmonaria* before starting ultrasonic acoustic emission (UAE) measurements.

Table S3. Relative water contents at which there was a significant increase of relative ultrasonic acoustic emissions (RWC_UAE_) on living and devitalized lobes of the lichens *Flavoparmelia caperata* and *Lobaria pulmonaria*.

Table S4. Relative water contents of the lichens *Flavoparmelia caperata* and *Lobaria pulmonaria* at the end of the rehydration protocol, and after the equilibrium with NaCl solution, and silica gel.

erae318_suppl_Supplementary_Figures_S1-S7_Tables_S1-S4

## Data Availability

The data that support the findings of this study are available from the corresponding author upon reasonable request.

## Abbreviations:

*F*_v_/*F*_m_: maximum quantum yield
IFW: initial fresh weight
LTSEM: low-temperature scanning electron microscopy
rcUAE: relative number of cumulative UAEs
RWC_tlp_: relative water content (RWC) at turgor loss point
UAE: ultrasonic acoustic emission
WL: water loss
Ψ_tlp_: water potential (Ψ) at turgor loss point
