# Supplementary material for: The sound of lichens: ultrasonic acoustic emissions during desiccation question cavitation events in the hyphae
Source: J Exp Bot. 2024 Jul 24;75(20):6579–92. doi: 10.1093/jxb/erae318 (PMC12536064; doi:10.1093/jxb/erae318)
Supplement: erae318_suppl_Supplementary_Figures_S1-S7_Tables_S1-S4 [file erae318_suppl_supplementary_figures_s1-s7_tables_s1-s4.pdf]

SUPPLEMENTARY DATA

Article title: The sound of lichens: ultrasonic acoustic emissions during desiccation question cavitation events in the hyphae

Authors: Enrico Boccato, Francesco Petruzzellis, César Daniel Bordenave, Andrea Nardini, Mauro Tretiach, Stefan Mayr, Fabio Candotto Carniel

Supplementary Fig. S1 Typical water potential (Ψ) isotherms (A, B), defined as the relationship between Ψ and water loss (WL), and the linear regression between 1/ Ψ and WL (C, D) for the lichens *Flavoparmelia caperata* (A, C) and *Lobaria pulmonaria* (B, D). Solid black lines (A, B) represent an exponential growth model (single, 2 parameters; adjusted $R^2 > 0.96$). Dashed black lines (c, d) represent the linear regression.

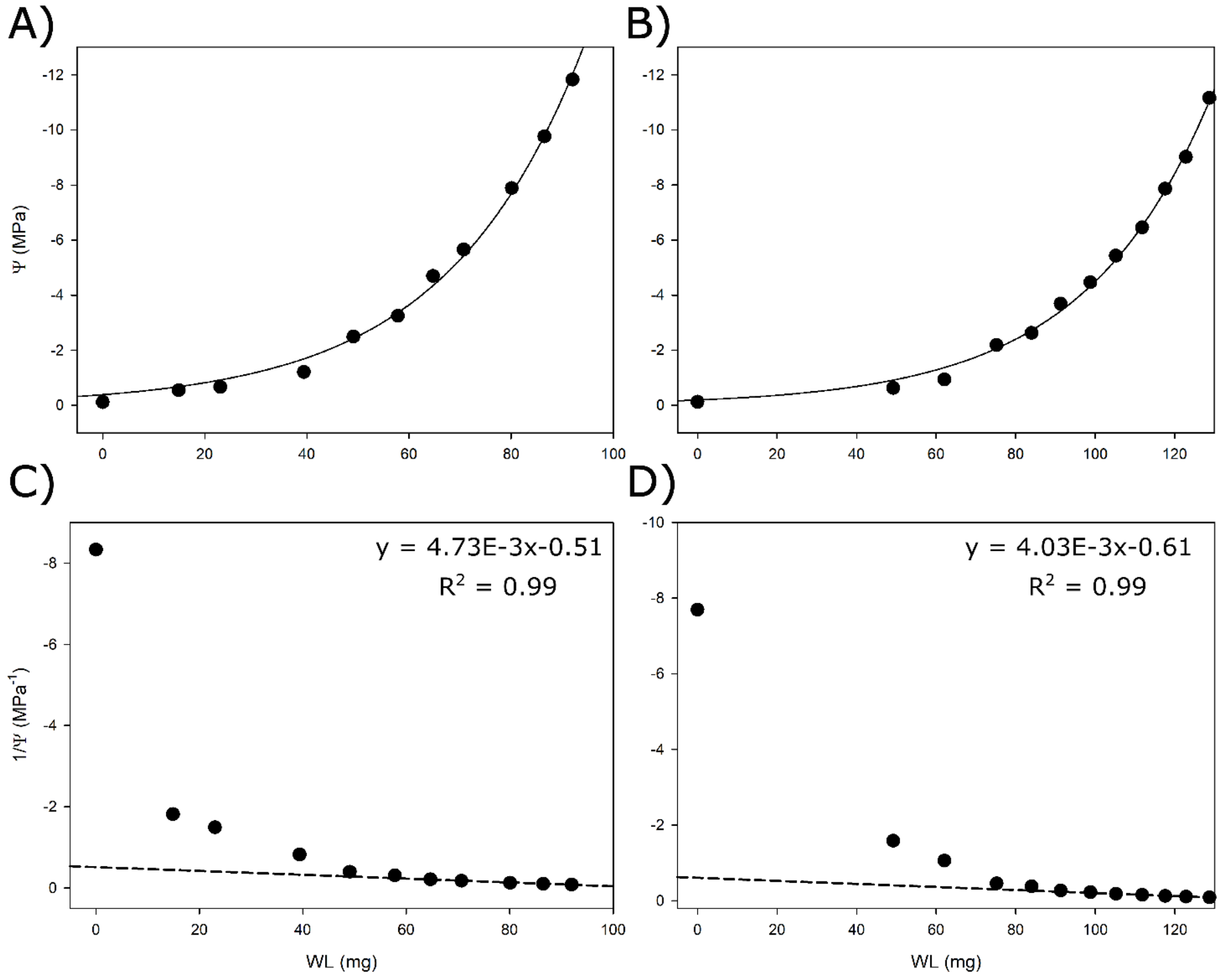

Supplementary Fig. S2 The setup used for the acquisition of the ultrasonic acoustic emissions (UAE). One set of samples (n = 4) was selected to continuously monitoring UAE (with conical weights on sensors); the other set of samples (n = 4) were prepared in the same way to reproduce the recording conditions, but the sensors were not attached to the UAE system. The schematic inset (made with Procreate®, Savage Interactive Pty Ltd.) represents a cross section of the lichen *Flavoparmelia caperata* together with the UAE setup: 1. UAE sensor; 2. Anagel™ Ultrasound gel; 3. Upper cortex; 4. Photobiont layer; 5. Medulla; 6. Lower cortex; 7. Worktable.

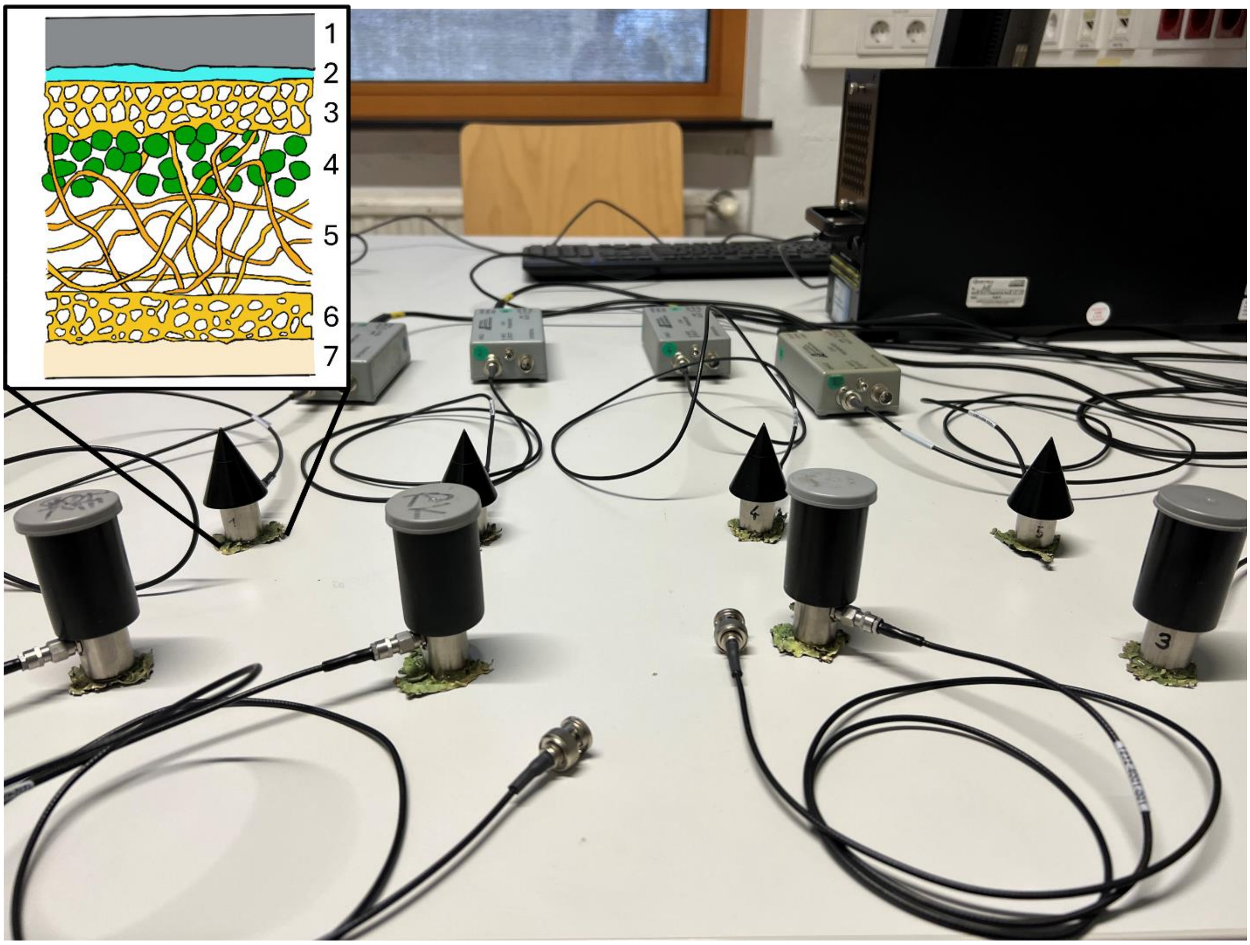

Supplementary Fig. S3 Relationship between time (s, x-axis) and absolute number of ultrasonic acoustic emissions (UAE; y-axis) in living (A) and devitalised (B) lobes of *Flavoparmelia caperata*, living (C) and devitalised (D) lobes of *Lobaria pulmonaria*, paper samples (E), and table (F). Different dot shapes are associated with different samples.

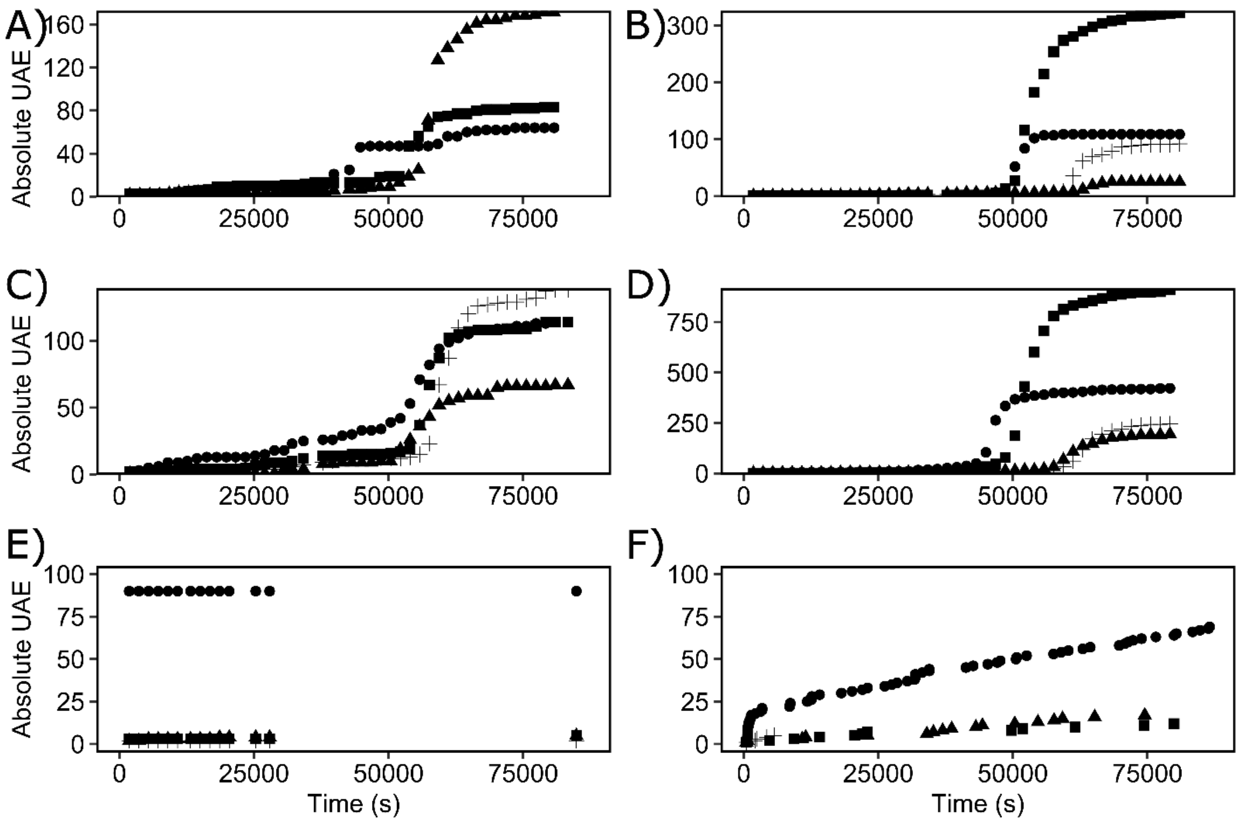

Supplementary Fig. S4 Relationship between relative water content (RWC; %, x-axis), amplitude (dB, right y-axis), and absolute energy (aJ, left y-axis) on devitalised lobes of the lichens *Flavoparmelia caperata* and *Lobaria pulmonaria*. Black dots indicate absolute energy values, while grey dots indicate amplitude values. Different shapes of the dots represent different samples.

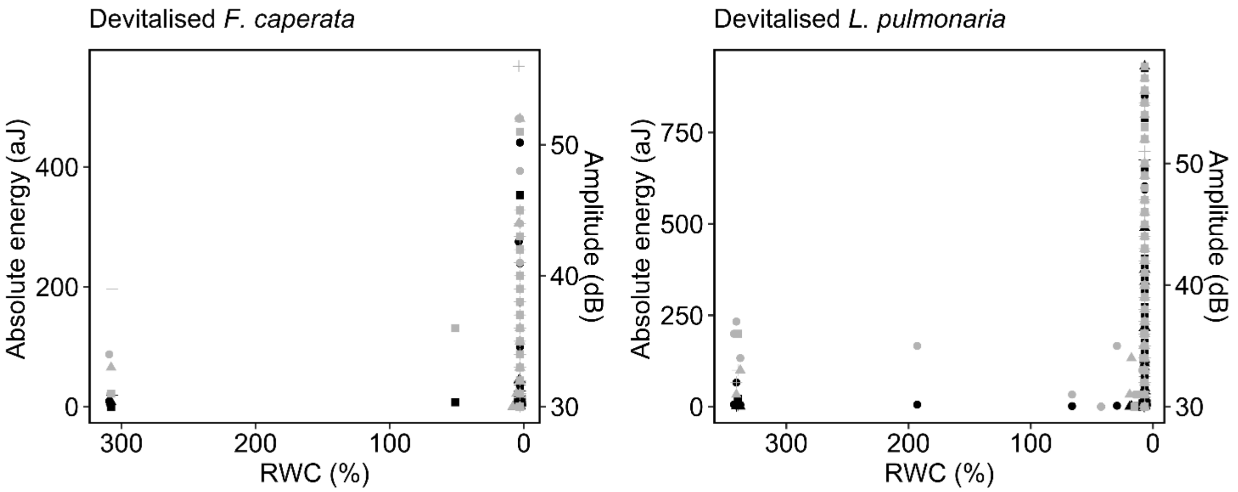

Supplementary Fig. S5 LTSEM micrographs of cryo-fractured lobes of *Flavoparmelia caperata* at different water contents, i.e. 167.1 ± 18.3% RWC (A, B), 16.7 ± 1.1% RWC (C, D), and 5.1 ± 0.9% RWC (E, F). Overview micrographs (A, C, E) and detail of each condition (B, D, F). White arrows indicate slightly shrunk cortical hyphae; white arrowheads indicate completely shrunk cortical hyphae. Scale bars A, C, E = 10 µm; B, D, F = 2 µm.

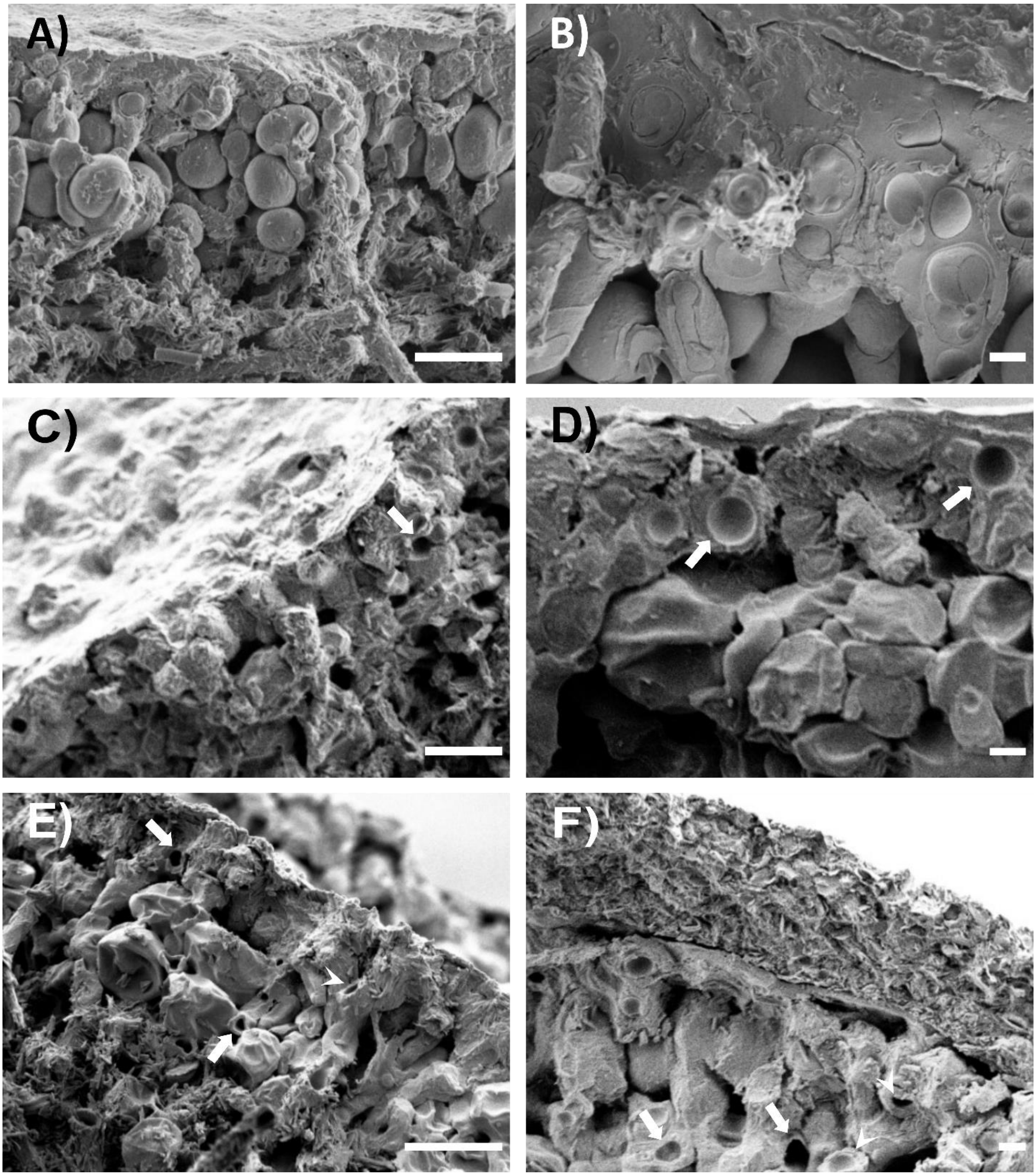

Supplementary Fig. S6 LTSEM micrographs of cryo-fractured lobes of *Lobaria pulmonaria* at different water contents, i.e. 151.4 ± 4.1% RWC (A, B), 16.4 ± 1.4% RWC (C, D), and 5.3 ± 0.5% RWC (E, F). Overview micrographs (A, C, E) and detail of each condition (B, D, F). White arrows indicate slightly shrunk cortical hyphae; white arrowheads indicate completely shrunk cortical hyphae. Scale bars A, C, E = 10 µm; B, D, F = 2 µm.

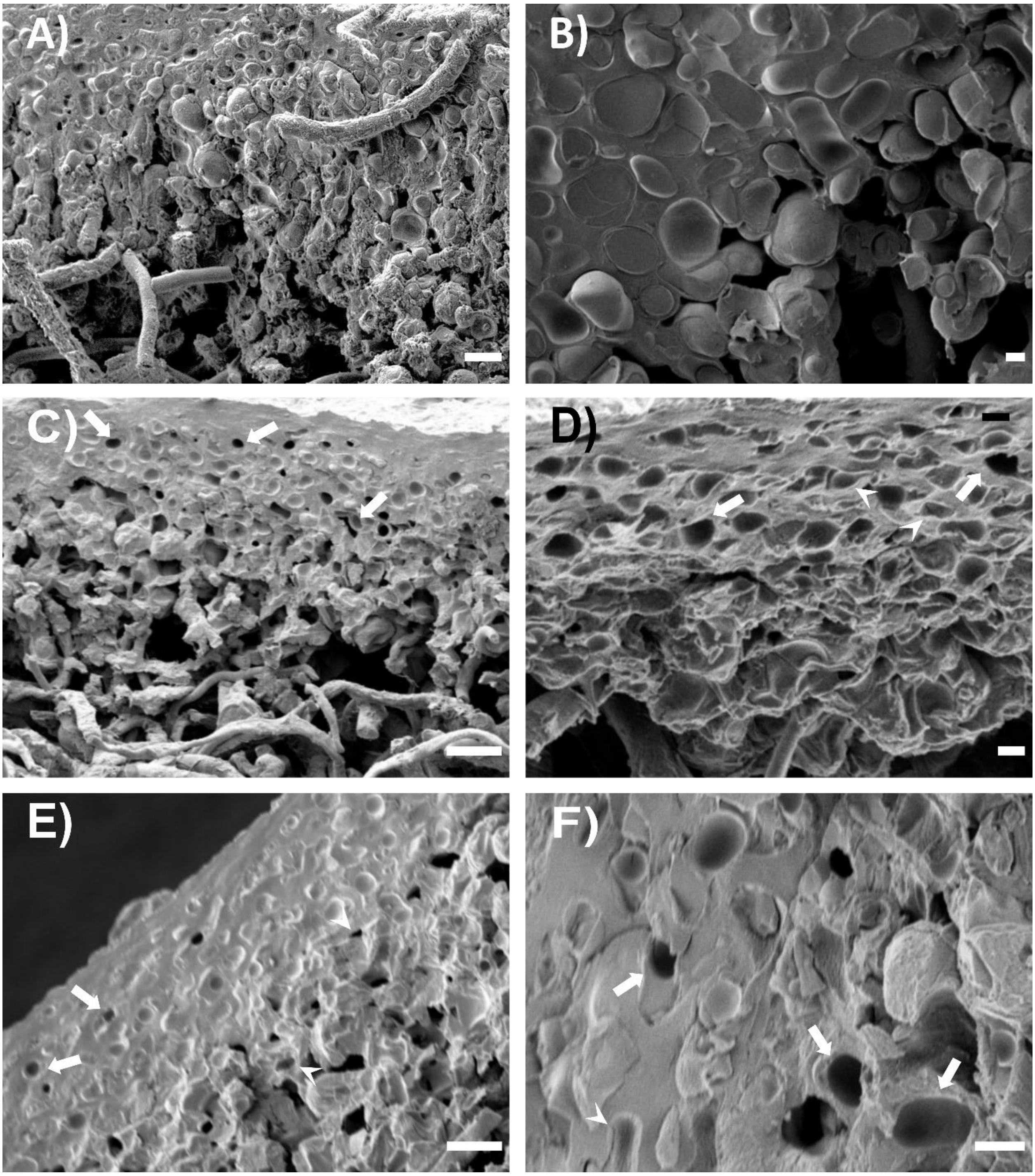

Supplementary Fig. S7 LTSEM micrographs of hyphae manifesting the onset of a "gas bubble" (white arrows) in a lobe of (A) *Flavoparmelia caperata* at 16.7 ± 1.1% RWC and (B) *Lobaria pulmonaria* at 5.3 ± 0.5% RWC. Fig. S7A is a detail of Fig. 6D. Scale bars = 2 µm.

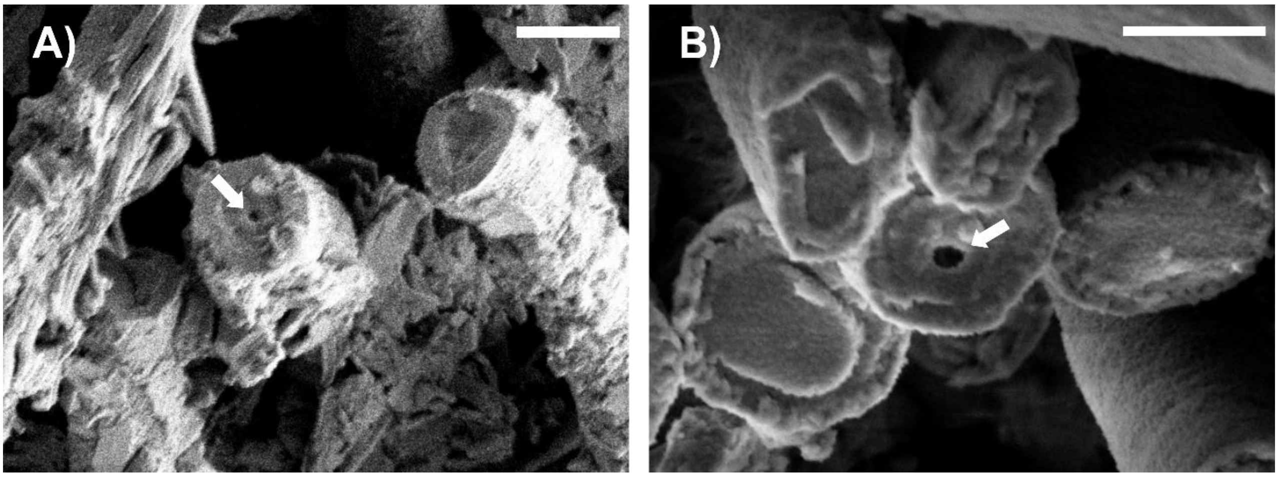

Supplementary Table S1 Water relation parameters derived by the elaboration of water potential (Ψ) isotherms of the lichens *Flavoparmelia caperata* and *Lobaria pulmonaria*. Water potential at turgor loss point (Ψ_{tlp}); relative water content at turgor loss point (RWC_{tlp}). Values are reported as means ± standard deviation (n = 6).

**Species**	**Ψ_{tlp} (MPa)**	**RWC_{tlp} (%)**
F. caperata	-6.0 ± 0.8	96 ± 12
L. pulmonaria	-7.4 ± 1.0	73 ± 25

Supplementary Table S2 PSII maximum quantum yield (F_v/F_m) on living and devitalised lobes of the lichens *Flavoparmelia caperata* and *Lobaria pulmonaria* before starting ultrasonic acoustic emissions measurements. Values are reported as means ± standard deviation (n = 8).

Species	F_v/F_m
F. caperata **(living)**	0.685 ± 0.064
F. caperata **(devitalised)**	0.107 ± 0.020
L. pulmonaria **(living)**	0.772 ± 0.010
L. pulmonaria **(devitalised)**	0.000 ± 0.000

Supplementary Table S3 Relative water contents at which there was a significant increase of relative ultrasonic acoustic emissions (RWC_{UAE}) on living and devitalised lobes of the lichens *Flavoparmelia caperata* and *Lobaria pulmonaria*. C.I.: bootstrap 5% and 95% confidence intervals, representing the lower and the upper limits of the C.I., respectively.

Species	RWC_{UAE}	Lower C.I. (5%)	Upper C.I. (95%)
***F. caperata* (living)**	7.9	7.6	8.1
***F. caperata* (devitalised)**	5.2	4.5	5.9
***L. pulmonaria* (living)**	6.7	6.1	7.2
***L. pulmonaria* (devitalised)**	8.4	7.9	8.9

Supplementary Table S4 Relative water contents of the lichens *Flavoparmelia caperata* and *Lobaria pulmonaria* at the end of the rehydration protocol (RWC control), and after the equilibrium with NaCl solution (RWC NaCl solution), and silica gel (RWC silica gel). Values are reported as means with ± standard deviation (n = 3).

Species	RWC control	RWC NaCl solution	RWC silica gel
F. caperata	167.1 ± 18.3	16.7 ± 1.1	5.1 ± 0.9
L. pulmonaria	151.4 ± 4.1	16.4 ± 1.4	5.3 ± 0.5
